# Adaptive Laboratory Evolution of Antibiotic Resistance Using Different Selection Regimes Lead to Similar Phenotypes and Genotypes

**DOI:** 10.3389/fmicb.2017.00816

**Published:** 2017-05-11

**Authors:** Leonie J. Jahn, Christian Munck, Mostafa M. H. Ellabaan, Morten O. A. Sommer

**Affiliations:** Novo Nordisk Foundation Center for Biosustainability, Technical University of DenmarkHørsholm, Denmark

**Keywords:** adaptive laboratory evolution, antibiotic resistance, selection pressure, collateral sensitivity, evolutionary constrains

## Abstract

Antibiotic resistance is a global threat to human health, wherefore it is crucial to study the mechanisms of antibiotic resistance as well as its emergence and dissemination. One way to analyze the acquisition of *de novo* mutations conferring antibiotic resistance is adaptive laboratory evolution. However, various evolution methods exist that utilize different population sizes, selection strengths, and bottlenecks. While evolution in increasing drug gradients guarantees high-level antibiotic resistance promising to identify the most potent resistance conferring mutations, other selection regimes are simpler to implement and therefore allow higher throughput. The specific regimen of adaptive evolution may have a profound impact on the adapted cell state. Indeed, substantial effects of the selection regime on the resulting geno- and phenotypes have been reported in the literature. In this study we compare the geno- and phenotypes of *Escherichia coli* after evolution to Amikacin, Piperacillin, and Tetracycline under four different selection regimes. Interestingly, key mutations that confer antibiotic resistance as well as phenotypic changes like collateral sensitivity and cross-resistance emerge independently of the selection regime. Yet, lineages that underwent evolution under mild selection displayed a growth advantage independently of the acquired level of antibiotic resistance compared to lineages adapted under maximal selection in a drug gradient. Our data suggests that even though different selection regimens result in subtle genotypic and phenotypic differences key adaptations appear independently of the selection regime.

## Introduction

Ecosystems continuously undergo changes in their physical and chemical properties resulting in shifts of ecological niches and living conditions (Hoffmann and Parsons, 1997; Elmqvist et al., 2003; Fine, 2015). Bacterial populations can respond to these environmental changes via both temporary and permanent adaptation. Temporary adaptation includes modulation of gene expression resulting in phenotypic changes, driven by changes in environmental signals, that are sensed by the bacteria (López-Maury et al., 2008). In contrast, selection of beneficial mutations or horizontal acquisition of advantageous genes represent permanent, genetic adaptations to a changed environment. Whether an environmental change is met by a temporary or a permanent adaptation largely depends on the strength and duration of the selection pressure. Substantial changes in the environment, as in the case of antibiotic treatment, can result in both temporary adaptations like metabolic alterations resulting in antibiotic persistent bacteria (Levin et al., 2014) and permanent adaptations that sometimes give rise to antibiotic resistant bacteria (Carroll et al., 2014; Dhawale and Rath, 2014).

This adaptive potential of microorganisms is increasingly explored in biotechnology by adaptive laboratory evolution (ALE) experiments (Blum et al., 2016). ALE can be utilized to improve production strains by increasing their tolerance to the metabolic product (Hu et al., 2016; Lennen, 2016), by activating latent pathways (Wang et al., 2016) or by enabling the utilization of non-native substrates (Lee and Palsson, 2010). In addition, ALE experiments can improve our understanding of fundamental evolutionary principles that might help us solve rising global challenges of undesirable adaptations like drug resistances in microbial pathogens (Anderson et al., 1950; Govan and Fyfe, 1978; Cohen et al., 1989; Donald and van Helden, 2009; Wensing et al., 2015), cancer (Riganti et al., 2015) or insect resistance toward pesticides (Georghiou, 2012). Usually, ALE experiments focus on the adaptation to specific physical or chemical factors such as temperature (Tenaillon et al., 2012; Sandberg et al., 2014) or antibiotic tolerance (Hegreness et al., 2008; Toprak et al., 2012; Lázár et al., 2014; Munck et al., 2014; Rodriguez de Evgrafov et al., 2015). Various ALE setups have been used to study similar environmental perturbations like exposure to different antibiotics (Hegreness et al., 2008; Toprak et al., 2012; Lázár et al., 2014; Munck et al., 2014; Rodriguez de Evgrafov et al., 2015), yet, the influence of the experimental setup on the resulting adaptations remains poorly understood.

In this study we use evolution of antibiotic resistance as a model for studying the impact of the experimental setup on evolved phenotypes and genotypes. Prior studies have evolved bacteria to high level antibiotic resistance using different methodologies, including gradients of increasing drug concentrations (Kim et al., 2014; Munck et al., 2014; Oz et al., 2014; Rodriguez de Evgrafov et al., 2015), step-wise exposure to antibiotics (Lázár et al., 2014) or gradual increase in drug concentration in a morbidostat (Toprak et al., 2012). These different approaches utilize varying selection pressures, population sizes, and bottlenecks–all known to impact evolution (Nei et al., 1975; Levin et al., 2000; Wahl et al., 2002; Charlesworth, 2009). The influence of the selection pressure on the resulting pheno- and genotypes has been assessed by comparing bacteria exposed to mild and strong selection in a gradient approach (Oz et al., 2014) as well as by challenging bacteria with drug increments, varying in the steepness of drug increase (Lindsey et al., 2013). Differences in the number of mutations, growth rate, and resistance phenotypes were detected dependent on the methodology used (Lindsey et al., 2013; Oz et al., 2014). While the gradient method applies maximal selection pressure resulting in rapid generation of highly resistant lineages, the increment approach requires fewer laboratory resources, allowing for investigations of larger number of replicates and conditions such as different antibiotics, without increased handling time. Yet, the rate of drug increase is fixed in the increment approach, which can result in low as well as too high selection pressure dependent on the adaptation level of the bacteria.

In this study, we compare different increment approaches, varying in the steepness of drug increase, with the gradient approach to investigate how the selection regime defined by the ALE methodology influences the resulting geno- and phenotypes.

## Materials and methods

### Laboratory adaptive evolution in drug gradients

*Escherichia coli* K12 (MG1655) was evolved for 14 days to three different antibiotics: Amikacin sulfate (AMK) (Sigma), Piperacillin sulfate (PIP) (Sigma), and Tetracycline hydrochloride (TET) (Sigma), covering three major classes of antibiotics, including both bactericidal and bacteriostatic drugs. The antibiotics were dissolved in water (10 mg/l) and the stock solutions were stored at −20°C. Four replicate lineages were evolved in parallel for each drug. 96-well plates (Almeco), containing 1 ml Mueller-Hinton broth II (MHBII) (Sigma) per well and a 2-fold antibiotic gradient in 10 dilutions, were prepared at the start of the experiment and stored at −20°C. The minimal inhibitory concentration (MIC) of the wild type, as defined by the European Committee on Antibiotic Susceptibility Testing (EUCAST), was located in the second well, allowing growth of the wild type in the first well under sub-inhibitory conditions (exact plate setup and drug concentrations are given in Supplementary Table 1). Plates were defrosted at the day of usage, pre-heated to 37°C, inoculated with 50 μl of freshly growing cells and incubated at ~900 r.p.m. and 37°C for 22 h. One hundred fifty microliters of each well were transferred into a 96-well microtiter plate and the optical density was measured at a wavelength of 600 nm (OD_600_) by an ELx808 Absorbance Reader (BioTek). Based on the OD measurement a cut-off value, that was the minimal growth that clearly set itself apart from the background growth, was chosen to define distinct growth for each drug (Supplementary Figure 1). An OD_600_ > 0.1 corresponding to ~8.0 × 10^7^ CFU/ml defined distinct growth for AMK and TET and an OD_600_ > 0.3 equivalent to about 2.4 × 10^8^ CFU/ml defined growth for PIP due to a background growth level of around OD_600_ = 0.18. Fifty microliters of the well with the highest drug concentration that showed distinct growth in the deep-well plate were used to inoculate a fresh gradient (exact OD_600_ values and corresponding drug concentrations of the well chosen for each transfer are given in Supplementary Table 2). Remaining cells in these wells were mixed to a final glycerol concentration of 20% and stored at −80°C. On each plate 16 wells served as negative control resulting in a total of 448 wells during the course of the experiment, of which 1% showed growth. For each lineage seven colonies were isolated for genomic and phenotypic characterization from the population that had been maintained for two passages at or above the clinical breakpoint as defined by EUCAST for the specific antibiotic. The clinical breakpoint is the drug concentration that is used as a cut-off value to classify pathogens as susceptible or resistant toward a specific drug (Turnidge and Paterson, 2007).

### Laboratory adaptive evolution in drug increments and media control

*E. coli* K12 (MG1655) was not only evolved in drug gradients but also to a daily relative increase of drug concentration. Three different approaches were used (exact drug concentrations for each day for the different increment approaches and drugs are given in Supplementary Table 3). The lineages in the “Increment 100” setting were exposed to a 100% increase in drug concentration. Under this regime the drug concentration was consequently doubled every day, applying a constantly strong selection pressure to the lineages. The clinical breakpoint was supposed to be reached after 7 days of the ALE experiment. “Increment 50” lineages were also exposed to a rather high environmental change rate by growth in a 50% higher drug concentration every day, reaching the clinical breakpoint on the 9th day of the experiment. The drug concentration was raised by 25% for the “Increment 25” lineages, allowing a mild selection and twice as much time to adapt to the clinical breakpoint concentration compared to the “Increment 100” lineages. Eight lineages were evolved in parallel in each setting to AMK, PIP, and TET. The experiment was designed that all experimental setups reached the MIC as defined by EUCAST at the 4th day. As control for media adaptations eight wild type lineages were evolved to the media without antibiotics. The MHBII antibiotic mixture was prepared in falcon tubes for each day, drug and experimental setup in the beginning of the experiment and stored at −20°C. The drug containing media was defrosted on the day of usage and pre-heated to 37°C. The lineages were grown in 1 ml MHBII and antibiotic in a 96-deep-well dish for 22 h at 37°C and ~900 r.p.m. 50 μl of cells were transferred every 22 h. The remaining cells were mixed to a final concentration of 20% glycerol and stored at −80°C. The adaptive evolution was stopped after 14 days when the “Increment 25” lineages had passed the clinical breakpoint. During the experiment ~3% of 1,152 negative controls showed growth. Cells were streaked on LB plates and identified by visual investigation as *E. coli*. All colonies looked identical, suggesting that there was no contamination. Once the adaptive evolution experiment was ended, lineages that were adapted to the clinical breakpoint were streaked on LB agar and seven isolated colonies were used for further analysis. If lineages died out before they had reached the clinical breakpoint, the last possible time point was chosen.

### IC_85_ determination

Isolated colonies were used to inoculate a 96-well microtiter plate containing 150 μl MHBII. About 10^5^ cells of an overnight culture were transferred with a 96-pin replicator to 10 dilutions of a 2-fold drug gradient spanning from 0.5 to 256 mg/l AMK and 0.25 to 128 mg/l of PIP or TET, respectively. For each lineage one isolated colony was tested in two technical replicates against all three antibiotics. Eight inoculated wells containing MHBII served as positive control while eight wells only filled with MHBII served as negative control. The plates were incubated at 37°C and 900 r.p.m. for 18 h and subsequently the OD_600_ was measured by an ELx808 Absorbance Reader (BioTek). The data was further analyzed using R (Team R Core, 2014). The average OD_600_ values of the negative controls were subtracted from all remaining OD_600_ values. Percent inhibition was calculated by subtraction of the OD_600_ values divided through the average of the OD_600_ values of the positive controls from 1.

(1) $$\begin{array}{l} Percent\;inhibition\\ =1-\frac{OD_{600}\;\left(growth\right)-\;OD_{600}\;\left(negative\;control\right)}{OD_{600}\;\left(positive\;control\right)-OD_{600}\;\left(negative\;control\right)} \end{array}$$

A dose-response curve was fitted to the values using a logistic model from the drc package (Ritz and Streibig, 2005), with x for the molar drug concentration and default values for the other variables, where *b* describes the steepness of the curve, *c* and *d* the lower and upper asymptotes and *e* the effective dose (Munck et al., 2014; Ritz et al., 2015):

(2) $$\begin{array}{l} f\left(x\left(b,\;c,\;d,\;e\right)\right)=\;c+\;\frac{\left(d-c\right)}{1+\text{exp}(b*\left(\log\left(x\right)-\log\left(e\right)\right)} \end{array}$$

Dose-response curves were plotted with the package ggplot (Wickham, 2009). The drug concentration causing 85% growth inhibition (IC_85_) was calculated with the inverted function, normalized to the wild type, and plotted grouped by drug using ggplot (Wickham, 2009). A non-parametric distribution of parallel lineages was observed for some experimental setups and drugs wherefore the non-parametric Kruskal-Wallis one-way analysis of variance was applied in R to detect significant (*P* < 0.05) differences between the four experimental setups adapted to each drug.

### Growth rate measurements

A 96-well microtiter plate, containing 200 μl MHBII per well, was inoculated with cells in exponential growth phase using a 96-pin replicator. All seven isolated colonies per lineages were included in the growth measurement. OD_600_ was measured in a ELx808 Absorbance Reader (BioTek) every 5 min for 10 h at 37°C and 650 r.p.m. The data was analyzed with R (Team R Core, 2014). The growth rate was calculated based on the steepest part of the growth curve during exponential growth. The doubling time was normalized to the wild type and plotted grouped by drug with the package ggplot (Wickham, 2009). To test whether the observed differences in growth rate were significant (*P* < 0.05) the non-parametric Kruskal-Wallis one-way analysis of variance was applied in R.

### Whole-genome sequencing

One isolated colony of each of the 92 lineages, that was also used to measure the IC_85_ and growth rate, was grown in LB and DNA was extracted with the A & A Genomic Mini kit (A&A Biotechnology). The DNA was sent to Macrogen, who prepared genomic libraries with the TruSeq DNA Nano (550 bp) kit from Illumina and sequenced them by Illumina MiSeq 300 bp paired ends.

### Identification of single nucleotide polymorphisms and small INDEL sequences

The reads were aligned to *E. coli* K12 U00096 reference genome with CLC Genomics Workbench (Escherichia coli Str. K-12 Substr, 2014). On average each base was covered at least 37.5 times (Supplementary Table 4). SNP and INDEL sites were determined with CLC Genomics Workbench. Only loci with a phred score of 30 at the variable position as well as at the three neighboring bases occurring at least with a frequency of 80% were included in the analysis. Single nucleotide polymorphisms (SNPs) that were detected in all lineages including the media adapted wild type were considered mutations that had occurred before the start of the experiment and were therefore excluded. If two lineages shared two identical SNPs they were considered cross-contaminated and one of them was excluded from further analysis.

### Detection of large deletions

Large deletions were identified with a workflow in CLC workbench. The reads of all genomes were assembled *de novo* and used as reference genomes to map the reads from one of the media adapted wild type strains. The reads from the media adapted strain that did not map to the *de novo* assembled antibiotic evolved genome were collected and also *de novo* assembled into contigs. Contigs larger than 1 kb with a coverage > 30 were considered large deletions.

### Detecting large DNA insertions by insertion elements

We downloaded both the complete genome of *E. coli* K12 MG1655, with accession U00096, and the corresponding ORFs from the NCBI Nucleotide Archive. The ORFs were then clustered together using cd-hit (Li and Godzik, 2006), allowing ORFs with at least 90% identity and coverage to be in the same cluster. The reads of the sequenced strains were filtered using the FASTX-Toolkit package and a minimum quality of 30. The quality-filtered reads were blasted against the clustered ORFs by setting the word_size to 10 and the e-value to 100 allowing accurate short sequence mapping. Reads mapping to at least two ORFs of different clusters with a minimum of 30 and a maximum of 70% coverage of each cluster were further considered potential large insertion reads. These reads were blasted against the whole genome to confirm that the two genes were not adjacent on the genome. Reads that covered the reference genome with more than 90% were expected to contain two adjacent genes, wherefore they were excluded from the analysis. It was further explored whether the remaining hits overlapped with insertion elements. Reads that aligned to one ORF annotated as insertion element were included for further analysis. Only insertion reads that were detected in more than 10 reads were considered as large DNA insertions.

### Identification of gene duplications

Gene duplications were identified with CLC Genomics Workbench using the coverage analysis tool by calling for regions with at least 700 bp of significantly (*P* < 0.001) increased coverage. The identified regions were mapped to the reference genome U00096 in R and genes that overlapped with at least 95% with the region of high coverage were identified as duplicated genes.

## Results

### Four different strategies of adaptive laboratory evolution

**Figure 1:**
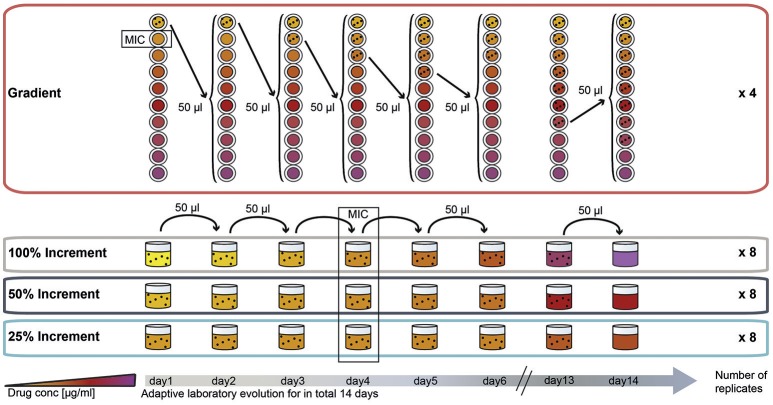
**Experimental design of the study: adaptation of ***E. coli*** K12 to three antibiotics following four adaptation protocols with different selection strengths**. The four selection systems are named “gradient,” “100% increment,” “50% increment,” and “25% increment.” In the gradient approach, cells were inoculated into a 2-fold, 10-step drug gradient and cells growing in the highest concentration were used for inoculation of a fresh gradient. In the increment approaches the drug concentration was raised with every transfer either by 100, 50, or 25%. The wild type minimal inhibitory concentration (MIC) was reached on the fourth day of the experiment for all increment lineages.

To compare the effect of the ALE methodology on final pheno- and genotypes we evolved *E. coli* to three different antibiotics using four different ALE strategies. The antibiotics chosen for this experiment represent three major groups of antibiotics. Two of the drugs, amikacin (AMK) and tetracycline (TET), target the ribosome with the former being bactericidal and the latter being bacteriostatic. The third drug, piperacillin (PIP), is a bactericidal drug targeting cell-wall biosynthesis. The four different selection regimes can be divided into two categories: (1) A gradient approach in which the population that tolerates the highest drug concentration is passed to a fresh drug gradient every 22 h; and (2) an increment approach in which the evolving population is passed every 22 h to a new drug concentration increased by fixed increments (Figure 1).

A 20-fold dilution of the gradient evolved populations was chosen based on a model by Wahl et al. (2002), to allow a high variation in the transferred population and to base fixation of mutations on the optimal adaptation rather then on limiting bottlenecks. According to Wahl et al. (2002) a 10-fold dilution of the population would be optimal but previous experiments in the laboratory showed a small increase in the inhibitory drug concentration for AMK and TET when a 10-fold dilution was used. To avoid an inoculum effect, which is a significant increase in the inhibitory concentration caused by a larger amount of organisms in the inoculum (Brook, 1989), we chose a 20-fold dilution (Supplementary Figure 2).

**Figure 2:**
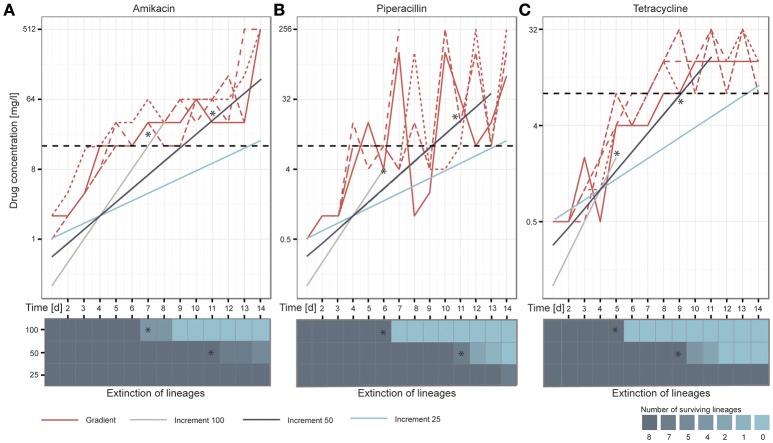
**Overview of the adaptation potential over time for each drug: (A)** amikacin, **(B)** piperacillin, and **(C)** tetracycline. The lineages adapted under the gradient method follow the maximal selection pressure wherefore it can be observed that lineages from the increment approaches (100, 50, and 25) die out when they exceed the natural selection maximum defined by the gradient approach, indicated with stars in the figure. Replicates are represented in different line styles. The clinical breakpoint is indicated with a dotted line in black. The heatmaps illustrate the extinction of lineages over time.

After 14 days of adaptive evolution in the gradient setup, the populations exposed to AMK tolerated on average 512 mg/l of the drug, corresponding to a 170-fold increase compared to the media adapted wild type (*P* = 2.89654E^−27^, student's *t*-test) (Figure 2A). The PIP evolved lineages grew in drug concentrations of 192 mg/l on day 14 of the ALE experiment, equal to a 80-fold increase compared to the media adapted wild type (*P* = 0.00109527, student's *t*-test). However, large oscillations in resistance were observed for the PIP evolved lineages during the course of the experiment (Figure 2B). This variation could be explained by an inoculum effect, which is more frequently observed for beta-lactam antibiotics (Eng et al., 1984, 1985; Brook, 1989). Lineages evolved to TET did not reach the same drug tolerance compared to AMK or PIP evolved lineages, but still grew in 15 mg/l TET, exceeding the media adapted wild type inhibitory concentration (IC) by 15 times (*P* = 2.04072E^−06^, student's *t*-test) (Figure 2C). These values are in accordance with previous findings where the IC_90_ values (the drug concentration at which growth of 90% of the population is inhibited) of isolated colonies were determined after 14 days of adaptive evolution in a gradient system using the same strain and drugs (Munck et al., 2014). Only PIP evolved lineages appear more resistant in the present study, which is attributed to the inoculum effect caused by the larger passaging volume.

In the increment approach three different rates of environmental change were tested (Figure 1), for which the selective pressure (e.g., antibiotic concentration) was increased by 100, 50, or 25% every day. Similar to the gradient approach the 100% increment setup applies a high selection pressure with the risk of exceeding the adaptive potential of the bacterium leading to extinction of the lineages (Figure 1).

### Extinction of increment lineages after exceeding the adaptation maximum defined by gradient lineages

We speculated that the increment-evolved lineages would die out when the drug concentrations they were exposed to exceeded the adaptation level of the gradient evolved lineages as a maximal selection pressure was applied to the gradient evolved lineages. Our observations during the ALE experiment support this hypothesis (Figures 2A–C). Whenever the antibiotic concentration of the drugs in the increment evolution experiments exceeded the maximal concentration of at least one of the gradient evolved lineages, some of the parallel increment lineages became extinct. For instance, the remaining 100% increment lineages adapted to AMK became extinct after they grew in 32 mg/l on day 8 of the experiment but one of the gradient adapted lineages only grew in 16 mg/l. All increment lineages died out when exposed to a drug concentration above the drug concentration to which all of the gradient lineages were adapted to (Figures 2A–C). For example all remaining increment 50% lineages died out when they passed an PIP concentration of ~32 mg/l on day 13 of the adaptive evolution experiment when lineages adapted in the gradient approach only grew in PIP concentrations of 8 mg/l. Since the antibiotic exposure for the 100 and 50% increment lineages exceeded the maximum evolutionary potential exhibited by the relevant gradient evolved lineages all 100 and 50% increment lineages went extinct before the end of the experiment (Figures 2A–C). Notably, only 4 out of the 24 lineages that were exposed to 100% increments reached the clinical breakpoint before extinction. A decrease in population density (OD_600_) often preceded extinction (Supplementary Figure 3). In contrast, all 50 and 25% lineages reached the clinical breakpoint. The ability to adapt appeared to be drug specific. The different adaptation potentials are reflected in the extinction of the increment lineages. For instance, 100 and 50% increment lineages died out later when adapted to AMK compared to TET (Figures 2A–C).

### Similar resistance levels can be accompanied by different fitness costs

We were interested in observing how the different rates of environmental change affected the final genotypes and phenotypes. By design, lineages would be adapted to different antibiotic concentrations at the end of the experiment, depending on the specific ALE approach. Accordingly, we decided to compare the lineages when they had reached the clinical breakpoint (defined by EUCAST, 2016). Gradient evolved lineages were analyzed at the time point when they had reached or exceeded the clinical breakpoint for 2 consecutive days. Increment evolved lineages were analyzed at the time point when the lineage had reached the clinical breakpoint. As most of the 100% increment lineages failed to reach the clinical breakpoint we excluded these lineages from the analysis.

**Figure 3:**
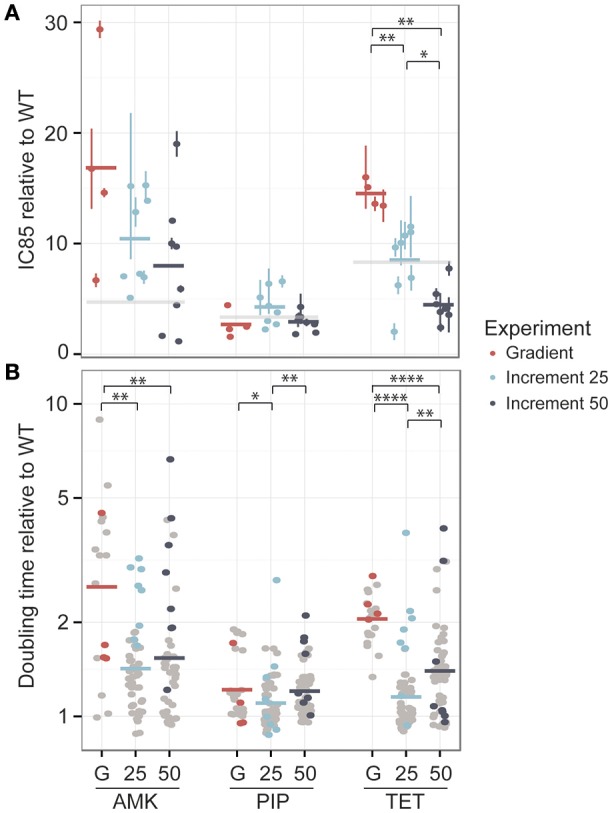
**Phenotypic changes in drug resistance and growth rate after the adaptive laboratory evolution experiment. (A)** The fold-increase of the IC_85_ compared to the wild type (WT) is displayed for each drug. The evolved resistance levels are not significantly different for amikacin (AMK) and piperacillin (PIP) across different experimental setups, which are referred to as G for the gradient approach, 25 for the 25% increment and 50 for the 50% increment method. However, strains adapted to tetracycline (TET) display significant changes in drug resistance. The clinical breakpoint normalized to fold-increase to the media adapted wild type (WT) of each drug is marked with a light gray panel. **(B)** The doubling time relative to the wild type is shown for the same isolated colony used for the IC_85_ determination as well as for six additional colonies, colored in gray. A significantly lower doubling time for the 25% increment lineages compared to the gradient evolved lineages can be observed for all drugs. ^*^P < 0.05, ^**^P < 0.01, ^***^P < 0.001, ^****^P < 0.0001.

One colony was obtained from each lineage at the time point where the population had reached the clinical breakpoint and the antibiotic tolerance was determined. The IC_85_ values were normalized to the average IC_85_ of the media adapted strains (Figure 3A).

For AMK and TET adapted strains the resistance level of the gradient evolved strains was above the clinical breakpoint (Figure 3A). In contrast, only one of the strains adapted to PIP was above the clinical breakpoint. High fluctuations in resistance level were observed in the PIP adapted lineages (Figure 2B) suggesting that an inoculum effect rather than real adaptation contributed to the population tolerance. The inoculum used in this study corresponded to about 10^8^ CFU/ml and did not indicate inoculum effect in previous experiments (Supplementary Figure 2). Yet, it is exceeding the reported CFU/ml concentration causing inoculum effect for PIP (Bryson and Brogden, 2012).

The resistance levels of the 25% increment evolved strains displayed a normal distribution around the clinical breakpoint for TET and PIP and were above the clinical breakpoint for AMK, whereas the 50% increment strains displayed a slightly lower tolerance (Figure 3A). However, when comparing gradient and increment adapted strains, only TET evolved strains showed a significant (*P* < 0.05 Kruskal-Wallis one-way analysis of variance) difference in their resistance levels (Figure 3A).

Many resistance-conferring mutations are known to confer a fitness cost, which can often be detected by a reduced growth rate (Linkevicius et al., 2013). Since the selection regime seems to influence the fitness of the resulting lineages (Lindsey et al., 2013), we measured the growth rate of the same isolated colonies that were used for the IC_85_ determination and an additional six isolated colonies for each lineage, resulting in 28 clones for the gradient approaches and 56 clones for the increment experiments (Figure 3B). Adaptation to AMK generally seemed to be connected with a reduced growth rate compared to the other drugs.

For all three drugs the 25% increment strains grew significantly (Kruskal-Wallis one-way analysis of variance *P* < 0.05) faster than the gradient adapted strains (Figure 3B). The growth advantage of the increment lineages could be due to a larger number of generations that they underwent compared to the gradient evolved lineages providing better opportunity for fitter mutants to outcompete resistant mutants with larger fitness costs and to accumulate compensatory mutations that can balance the fitness costs of resistance conferring mutations. However, the time that a population was evolved for and the doubling time are not significantly correlated (*R* = −0.019, Pearson's product-moment correlation coefficient, *P* = 0.71). Accordingly, it is likely that the shorter doubling time of the increment lineages is due to a lower selection pressure toward drug resistance, resulting in an increased selection for high growth rate. This finding is in line with previous studies reporting that *E. coli* lineages evolved to rifampicin under sudden drug increase have a significantly reduced growth rate compared to lineages evolved to more gradual drug increases (Lindsey et al., 2013) and that *E. coli* lineages adapted to 22 different antibiotics under mild selection have an elevated growth rate compared to lineages evolved under strong selection regimes (Oz et al., 2014). Yet, no correlation was found between the resistance level and the growth rate suggesting that a mutation that confers high-level resistance is not necessarily linked to a high fitness cost and *vice versa* (Supplementary Figure 4).

### Different selection regimes do not substantially influence collateral sensitivity and cross-resistance phenotype

**Figure 4:**
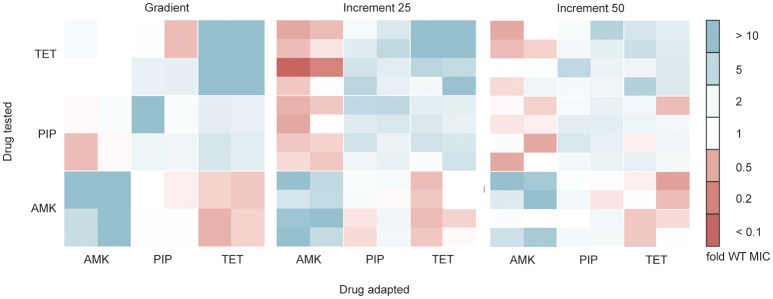
**Cross-resistance and collateral sensitivity phenotypes of the gradient, 25 and 50% increment evolved strains**. Fold increase (blue) or decrease (red) of the IC_85_ compared to the wild type (WT) is indicated for each individual strain. The collateral sensitivity as well as cross-resistance is similar across experimental setups. Collateral sensitivity toward tetracycline (TET) of strains adapted to amikacin (AMK) under the 25% increment approach is elevated (*P* < 0.05) compared to strains evolved to the drug gradient. The cross-resistance of strains evolved to piperacillin (PIP) tested against TET is increased (*P* < 0.05) in the 25% increment adapted strains compared to the gradient evolved strains.

Antibiotic resistant bacteria often show cross-resistance to similar drugs (Szybalski and Bryson, 1952). Interestingly, increased susceptibility toward other antibiotics can also frequently be observed (Szybalski and Bryson, 1952; Imamovic and Sommer, 2013; Lázár et al., 2014; Munck et al., 2014), a phenomenon commonly referred to as collateral sensitivity. In order to test if the cross-resistance and collateral sensitivity phenotype is influenced by the selection regime we determined the drug resistance profiles for each evolved strain toward each of the three drugs tested (Figure 4). All lineages adapted to AMK showed collateral sensitivity toward PIP. However, the gradient and 25% increment AMK adapted strains differed in their collateral sensitivity toward TET (*P* < 0.05, Kruskal-Wallis one-way) (Figure 4). It should be noted that the end point of the gradient adapted AMK lineages showed collateral sensitivity toward TET in accordance with previous studies (Munck et al., 2014), suggesting that the number of generations that a lineage was allowed to undergo before testing the collateral sensitivity was important. Isolates from gradient and increment lineages all showed collateral sensitivity against AMK when evolved to TET and cross-resistance between PIP and TET (Figure 4). However, strains adapted to PIP in the gradient approach were slightly less resistant (*P* < 0.05, Kruskal-Wallis one-way) to TET than the strains adapted in the 25% increment approaches (Figure 4). While, Oz et al. (2014) highlights differences in collateral sensitivity in mildly and strongly selected lineages, our results are in line with findings by Lázár et al. (2014) suggesting that phenotypic similarities dominate over differences.

Overall it can be concluded that the cross-resistance was very similar between gradient and increment approaches. The main phenotypic difference between gradient and increment evolved lineages is a slower growth rate of the gradient evolved lineages.

### Genotypes of lineages adapted under different selection pressures overlap

The strains used for IC_85_ determination and growth rate measurements were sequenced in order to uncover the underlying genetic changes. We identified a total of 173 mutations across 92 sequenced strains (Supplementary Table 5). Large insertions and deletions made up 26.5% of the total number of mutations (Supplementary Figure 5A). These larger genetic rearrangements are frequently overlooked but can play important roles in the genetic adaptation process. Two of the eight parallel strains adapted in a 25% increment to AMK have three identical SNPs in common, suggesting potential cross-contamination between the lineages. Therefore, only one of the strains was used for the following analysis.

On average we identified about two mutations in each strain across the different experiments (Supplementary Figure 5B). Even though the 25% increment lineages were evolved for more generations until they reached the clinical breakpoint there was no significant difference in the number of mutations between experimental setups (*P* > 0.5, Students *t*-test) and the number of mutations in the sequenced isolates did not correlate significantly with the number of generations (*R* = 0.19, Pearson's correlation, *P* = 0.097).

**Figure 5:**
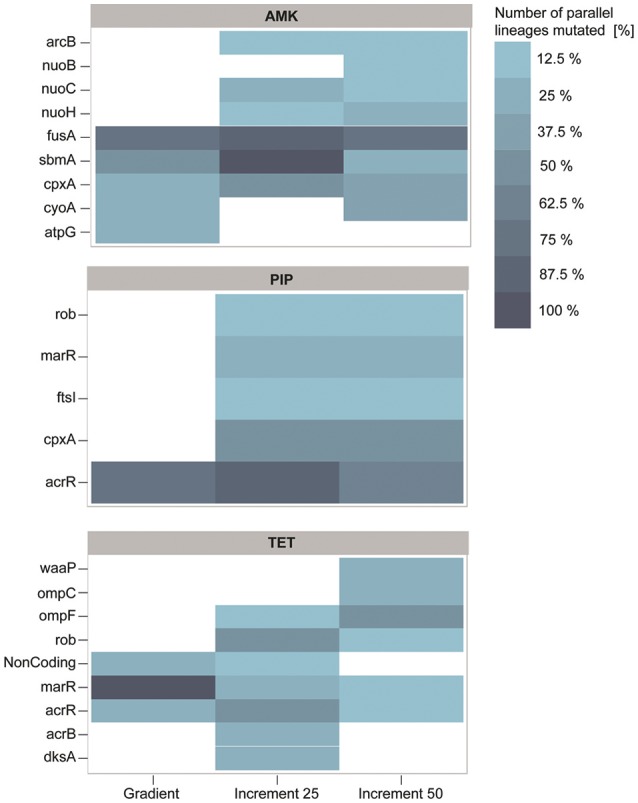
**Genetic adaptations to the antibiotics amikacin (AMK), piperacillin (PIP), and tetracycline (TET)**. The color represents the number of strains in percent that harbor a mutation, identified in at least two independent lineages. Almost all mutations identified in the gradient approach are also present in at least one of the increment strains. PIP and TET evolved lineages display similar genotypes with mutations predominantly affecting the mar phenotype.

Whether a mutation confers resistance, compensates fitness costs of other mutations or hitchhikes with a resistance mutation is difficult to determine without re-introducing specific mutations alone and in combinations into the non-evolved wild type. However, if a gene is mutated in more than one independent strain it is likely that the mutation was selected for (Lieberman et al., 2011; Yang et al., 2011; Sandberg et al., 2014). We filtered our dataset according to this criterion and found that 88.8% of genes mutated in the gradient evolved strains were also mutated in the increment strains (Figure 5). Except mutations in the ATP synthase gamma chain (*atpG*) and the cytochrome bo(3) ubiquinol oxidase subunit 2 (*cyoA*), all mutated genes of the gradient strains in the filtered dataset have also been found to be mutated in the 25% increment strains (Figure 5). The clones carrying one of the two mutations have an average doubling time that is four times higher than the wild type and twice as high as the average of all strains adapted to AMK. Therefore, it is likely that these mutations come with a high fitness cost, and accordingly were not fixed in the 25% increment lineages.

Interestingly, the increment-adapted strains carried not only most of the mutations found in the gradient adapted strains, but also many mutations that were solely identified in the increment strains (Figure 5). Such mutated genes are for example *nuoB, nuoC*, and *nuoH*, subunits of the NADH-quinone oxidreductases that shuttle electrons from NADH to quinones in the respiratory chain that have been identified to confer resistance toward AMK in previous studies (Kohanski et al., 2007; Schurek et al., 2008; Girgis et al., 2009; Wong et al., 2014). In addition, these mutations were linked to the collateral-sensitivity phenotypes of aminoglycosides toward many other classes of antibiotics (Lázár et al., 2014), suggesting that mutations in these genes are relevant for the collateral sensitivity phenotype toward TET, that was not observed in the gradient lineages when they reached the clinical breakpoint, but only in strains isolated from the end point of the gradient evolved lineages.

Mutations in the genes *fusA, sbmA* as well as in two different two component systems, *cpxRA* and *arcAB* appeared to be the dominating mutations in all strains adapted to AMK (Figure 5). All mutations have been previously linked to AMK resistance (Laviña et al., 1986; Busse et al., 1992; Johanson and Hughes, 1994; Salomón and Farías, 1995; Macvanin and Hughes, 2005; Kohanski et al., 2008, 2010; Pena-Miller et al., 2013; Lázár et al., 2014; Munck et al., 2014). Mutations in the elongation factor G encoding gene *fusA* have been shown to result in collateral sensitivity toward beta-lactam antibiotics, as observed in this study for PIP (Macvanin and Hughes, 2005).

*acrR* was found to be the predominantly mutated gene in the PIP evolved lineages regardless of the experimental setup (Figure 5). Mutations in *acrR* as well as in *marR* and *rob*, also identified to be mutated in the increment strains adapted to PIP, lead to the multiple antibiotic resistance (mar) phenotype, which was described to confer resistance toward a variety of drugs including beta-lactam antibiotics and tetracyclines. This finding explains the cross-resistance observed for TET and PIP evolved lineages in this experiment (George and Levy, 1983; Cohen et al., 1989; Ariza et al., 1995; Maneewannakul and Levy, 1996; Oethinger et al., 1998). The lack of mutations in *marR* and *rob* in the gradient adapted strains might account for the difference in cross-resistance toward TET compared to the 25% increment adapted strains. However, it can be speculated that these mutations would also occur in a gradient system if the inoculum effect can be avoided, since they were observed previously in an experiment following the gradient approach (Munck et al., 2014). Another frequently observed mutation in PIP adapted strains affects the drug target, the peptidoglycan synthase *ftsI* (penicillin-binding protein 3) (Figure 5) (Matic et al., 2003; Blázquez et al., 2006). Interestingly, mutations in *cpxA* were solely found in 25 and 50% increment strains adapted to PIP (Figure 5). Mutations in this gene can confer up to 2-fold increases in resistance to beta-lactam antibiotics (Srinivasan et al., 2012; Bernal, 2014). Since a 2-fold increase in drug resistance is moderately low, it can explain why the mutation was only found in 25 and 50% increment lineages that were exposed to low antibiotic concentrations and why it was not identified in the gradient or 100% increment lineages.

The lineages adapted to TET showed, similar to PIP, mutations in genes belonging to the mar phenotype (Figure 5). In addition to mutations belonging to the mar phenotype, two other mutated genes, *dksA* and *waaP*, were identified which were previously only indirectly linked to antibiotic susceptibility (Yethon et al., 1998; Yethon and Whitfield, 2001; Hansen et al., 2008; Tamae et al., 2008; Liu et al., 2010). Interestingly, the only gene duplications observed in this experiment were all found in three different lineages in the 50% increment strains adapted to TET. Two genes, *yicS* and *yibT*, with uncharacterized gene products, were duplicated as well as *phoU*, whose deletion mutant was more susceptible toward antibiotics suggesting a potential role in antibiotic tolerance (Li and Zhang, 2007).

The significant difference in resistance between gradient and increment evolved lineages might be explained by the abundance of mutations in *marR*. Almost all strains adapted under the gradient approach carry a mutation in *marR*, whereas this genetic change was only observed in a few lineages evolved in the increment regime (Figure 5). Lineages harboring mutated *marR* were overall 15% more resistant to TET than all TET adapted lineages on average. However, they also had an increased doubling time by ~27% compared to the average. Δ*marR* mutants were previously linked to an impaired fitness (Marcusson et al., 2009), suggesting that mutations in *marR* are more likely to dominate a population under strong selection.

**Figure 6:**
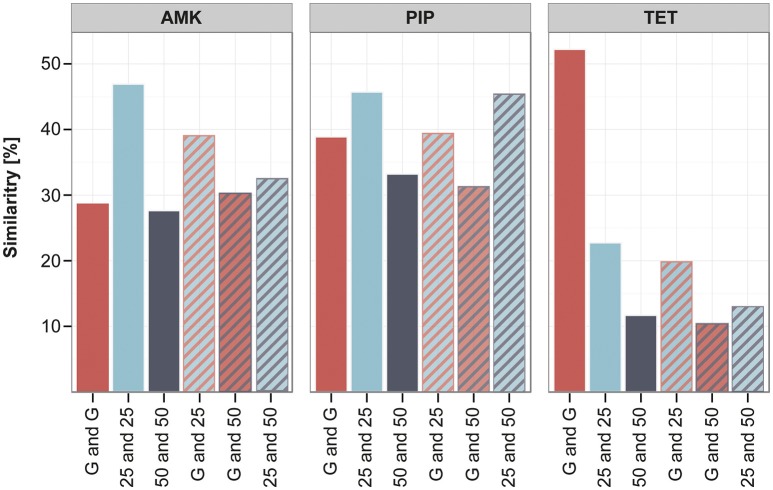
**Similarity of lineages evolved to different selection strengths**. The similarity between different strains was calculated in pairwise comparisons by analyzing the percentage of genes that were mutated in both strains compared to the total number of mutations found in the two strains. The average of the pairwise similarity is displayed for different groups of strains: either within one experimental setup like the gradient (G) (red) or the increment approaches (25 and 50) (blue and gray) or between those experimental setups (stripes), comparing similarity of the gradient and increment lineages or the increment approaches with each other.

The clones adapted under the gradient approach seem to have fewer mutations in the filtered data set in comparison to the increment lineages. However, they often carry mutations that were only detected once in the whole experiment (Supplementary Table 5), therefore the gradient adapted lineages display a higher diversity in unique mutations. In order to quantify the similarity and dissimilarity between genotypes in the different experimental setups, we did a pairwise comparison between all strains using the unfiltered data set. The overlap of mutated genes between the pairs was calculated in percent of the total number of mutated genes found in the two strains. We chose to analyze the similarity on the gene level and not on SNP or gene family level, since it was suggested as appropriate measure to detect parallel or convergent evolution (Achaz et al., 2014). The genetic similarity within the gradient evolved replicates was on average around 30–50% (Figure 6). The strains adapted under the 25 and 50% increments were about 45 and 30% similar to each other when adapted to AMK and PIP and only around 20 and 10% alike when evolved to TET (Figure 6). Interestingly, the genetic similarity of strains evolved using different selection regimens was comparable to the similarity within replicates from the same selection regimen (Figure 6). The similarity of the gradient and the 25% increment strains was maximal about 3% below and 12% above the group internal similarity of either the gradient or the increment 25 strains (Figure 6). This result underlines that the genetic similarity between the different selection regimens is similar to the genetic similarity observed between parallel lineages that were evolved under identical conditions.

## Discussion

In this study we analyzed the impact of the selection regimen in different ALE methodologies on the resulting phenotypes and genotypes. As expected, the rate of environmental change is a crucial parameter for the extinction of populations. Environmental changes, that exceed the adaptation capability of an organism or that allow too little time for adaptation, result usually in extinction. A decrease of the OD often preceded extinction of lineages, indicating that the mean fitness of the population was reduced preceding extinction (Lynch and Lande, 1993). The reduction in population size also lowered the number of cells transferred to the next drug concentration, reducing the genetic variability (Frankham, 2005; Bell and Collins, 2008). Under these conditions the mean population fitness is impaired and can only be enhanced through a lowered environmental change rate (Lynch and Lande, 1993). In our case the constantly high rate of environmental change in the 100 and 50% increment lineages led to extinction of the evolving population. We observed an evolutionary limit for adaptation that was defined by the gradient evolved lineages. If the rate of environmental change of the 50 and 100% increment lineages exceeded this maximum, strains became extinct. These findings can be implemented in the design of an ALE experiment for industrial purposes where the extinction of lineages should typically be prevented. Therefore, it can be suggested to either use a milder rate of environmental change or a higher number of replicates in order to compensate for lineage extinction.

We expected, that the gradient evolved lineages would indicate the evolutionary capacity of *E. coli* to adapt to a certain drug as we constantly applied maximal selection pressure in the gradient setup. However, in case of Piperacillin we observed large oscillations of the drug tolerance in all parallel evolving populations during the 14 days of the adaptive evolution experiment. The inoculum varied during the cause of the experiment and sometimes exceeded the inoculum that was used for the initial test that did not suggest inoculum effect for any of the three drugs. In addition, beta-lactam antibiotics are known to be more prone to cause inoculum effect (Eng et al., 1985; Brook, 1989), wherefore we conclude that inoculum effect is the likeliest explanation for the oscillations. Yet, other scenarios such as (1) clonal interference (de Visser and Rozen, 2006), where several mutations conferring resistance, tolerance or growth advantageous compete against each other, (2) disruptive frequency dependent selection (Levin et al., 1988), where only common mutations are fixed in the population or (3) phenotypic tolerance, that temporarily allows bacteria to survive in antibiotic concentrations without conferring resistance (Brauner et al., 2016) could potentially account for the oscillations as well.

Once a given phenotypic level has been reached, different paths of selection lead to similar phenotypes and genotypes. The similar outcomes of variations in selection pressure strength can be explained by the concept of evolutionary constrains (Losos, 2011). Due to a limited number of accessible changes to adapt to a certain selection pressure, evolution is biased toward these mutations, resulting in similar changes in organisms exposed to comparable environments (Losos, 2011).

We found that differing selection strength, applied through a daily increase in drug concentration by 25% or a drug gradient, follows similar evolutionary trajectories, resulting in similar phenotypes and genotypes. The cross-resistance and collateral sensitivity patterns appear very similar between both approaches. This is in line with previous findings, where lineages evolved to sub-inhibitory drug concentrations were compared to those adapted under strong selection (Lázár et al., 2014). Yet, also opposing results have been reported, showing differences in mildly and strongly selected lineages (Oz et al., 2014). However, the cross-resistance and collateral sensitivity differences observed by Oz et al. were mildly connected to the final resistance level of the strain to the adapted drug, which could account for these phenotypic differences (Oz et al., 2014). To limit these confounding factors we sought to investigate lineages as they had reached similar resistance levels toward the adapted drug. The phenotypic differences observed by Oz et al. were explained by genotypic variations. A larger number of mutations was reported for the strongly selected lineages including a higher variety of mutations concerning the drug targets. These findings contrast other studies claiming that the stronger the selection pressure, the less evolutionary trajectories are open to meet the adaptation requirements, resulting in fewer but more impactful mutations in strongly evolved lineages (Barrick and Lenski, 2013; Lindsey et al., 2013) and may result from differences in the finally evolved phenotypes of the strains compared to the present study. In our study we find a similar number of mutations in strongly and mildly adapted lineages. However, when filtering our genomic data for recurring mutations, we find a smaller number of mutations in the gradient evolved lineages compared to the increment adapted lineages, suggesting that fewer resistance conferring mutations are selected for under strong selection. In spite of this small difference, the genetic similarity between replicates within one selection approach is comparable to the similarity between different selection approaches. These findings suggest that ALE experiments conducted with varying protocols are indeed comparable. Accordingly, one doesn't loose genetic or phenotypic information when using a high throughput applicable increment approach compared to gradient systems.

Nonetheless, one important difference with implications for ALE experiments was detected in this study: Lineages adapted with the 25% increment method consistently displayed a higher growth rate compared to the growth rate of isolates from lineages evolved to stronger selection pressure. The growth advantage of strains adapted with the 25% increment method can be explained by the mild selection regime that increases the selection pressure on fitness rather than on highest resistance levels, possibly selecting for mutations that compensate fitness costs or generally increase the fitness. When the sequencing data was filtered to consider only mutations that have been detected in more than two individual lineages, clones adapted in 25% increments carried almost all mutations that were found in the gradient lineages. These mutations are therefore likely to be most important for the resistance phenotype. A number of additional mutations were solely identified in the increment lineages. These mutations may confer resistance at a lower fitness cost or balance out fitness disadvantages of the resistance conferring mutations. Depending on the aim of the ALE experiment, attention should be paid to the impact of the method on the growth rate. Especially, if the ALE experiment is conducted to improve a biotechnological production strain, the growth rate of the evolved strains can be an important factor to select for (O'Brien et al., 2016).

In case of antibiotic resistance, ALE experiments can be useful to explore the evolutionary potential of a species to develop antibiotic resistance. Some of the mutated genes that were identified in this and previous studies, like the *marR, rob*, or *acrAB* loci, have been reported to carry mutations also in clinical isolates (Oethinger et al., 1998; Sáenz et al., 2004; Buffet-Bataillon et al., 2012). However, other mutations identified by ALE experiments do not occur in natural environments. Regardless, ALE experiments can provide a better understanding of the genetic and phenotypic flexibility of the organism and its adaptation potential in response to challenging environmental conditions.

We were interested to see if both, the gradient as well as the increment approach, mimic natural occurring resistance evolution similarly. To investigate the generality of the identified SNPs we mined all sequenced *E. coli* genomes for non-conservative SNPs in coding regions. More than 50% of the mutated genes in both the increment 25% and gradient approach and all of the genes that the approaches have in common overlap with the mutated genes in the database (Supplementary Figure 6), indicating that both methodologies simulate natural resistance evolution to a similar extend. Obviously, it remains to be clarified if these SNPs actually confer resistance or other advantages in the host environment. Yet, the occurrence of such mutations in both natural isolates as well as laboratory-evolved populations suggests a biological importance.

Our results demonstrate that key adaptations to AMK, PIP and TET in *E. coli* are independent of the selection regimen and that mutations that robustly occur regardless of the selection regime or ALE methodology are also more likely to be selected for in the clinic than mutations that are selected only under very specific selection conditions.

## Author contributions

MS, CM, and LJ planned the project and designed the experiments. LJ conducted the experiments and carried out the data analysis with help from CM. ME contributed by identifying large insertion sequences and by writing the Material and Methods section about his analysis. LJ wrote the manuscript, which was critically reviewed by CM and MS.

## Funding

This work was supported by the European Union's Horizon 2020 research and innovation program under the Marie Sklodowska-Curie grant agreement No 642738, MetaRNA as well from the European Union's Horizon 2020 (ERC-2014-StG) under grant agreement 638902, LimitMDR. In addition, funding was received from the Danish Free Research Council and from the Novo Nordisk Foundation through the Novo Nordisk Foundation Center for Biosustainability.

### Conflict of interest statement

The authors declare that the research was conducted in the absence of any commercial or financial relationships that could be construed as a potential conflict of interest.
