# Supplementary material for: Adaptive Laboratory Evolution of Antibiotic Resistance Using Different Selection Regimes Lead to Similar Phenotypes and Genotypes
Source: Front Microbiol. 2017 May 11;8:816. doi: 10.3389/fmicb.2017.00816 (PMC5425606; doi:10.3389/fmicb.2017.00816)
Supplement: Supplementary file 1 [file DataSheet1.pdf]

Supplementary figures

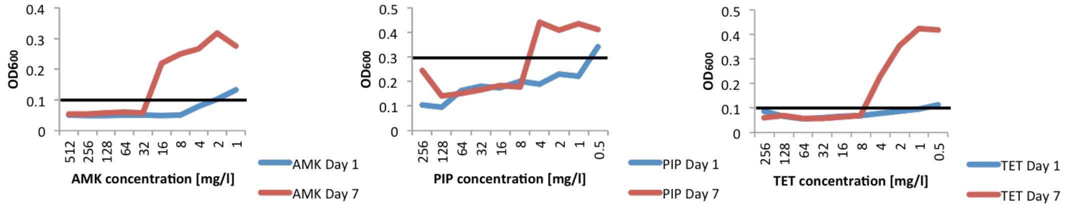

SI Figure 1: Growth in the different drug concentrations of the gradient. For each drug, amikacin (AMK), piperacillin (PIP) and tetracycline (TET) OD_{600} values for each drug concentration in the 10 dilutions of a two-fold gradient are shown in order to illustrate how we chose our cut-off value to define distinct growth. As it can be seen higher OD_{600} values are reached during the course of the experiment but OD_{600} values in the beginning are fairly low. In order to be able to use the same cut off value for one drug for the entire experiment we chose the lowest possible OD_{600} value that shows distinct growth compared to the background we chose $OD_{600} > 0.1$ for AMK and TET and $OD_{600} > 0.3$ for PIP.

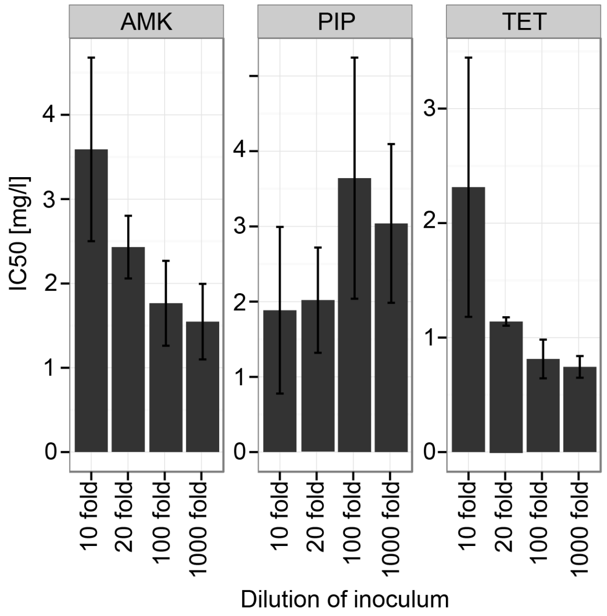

SI Figure 2: Effect of the dilution or inoculum size on the IC_{50}. Freshly growing MG1655 was inoculated into 10 dilutions of a two-fold gradient for three different drugs: amikacin (AMK) with an EUCAST MIC of 2 mg/l, piperacillin (PIP) with an EUCAST MIC of 1-2 mg/l and tetracycline (TET) with an EUCAST MIC of 1 mg/l. From the well with the highest drug concentration that showed growth (as defined in

Materials & Methods) new gradients were inoculated in triplicates with 10, 20, 100 and 1000 fold dilution. The IC_{50} values of these gradients were determined and are presented in this graph. A 10-fold dilution seems to elevate the IC_{50} values for AMK and TET, suggesting inoculum effect. Therefore, a 20-fold dilution was chosen for the adaptive laboratory evolution experiment.

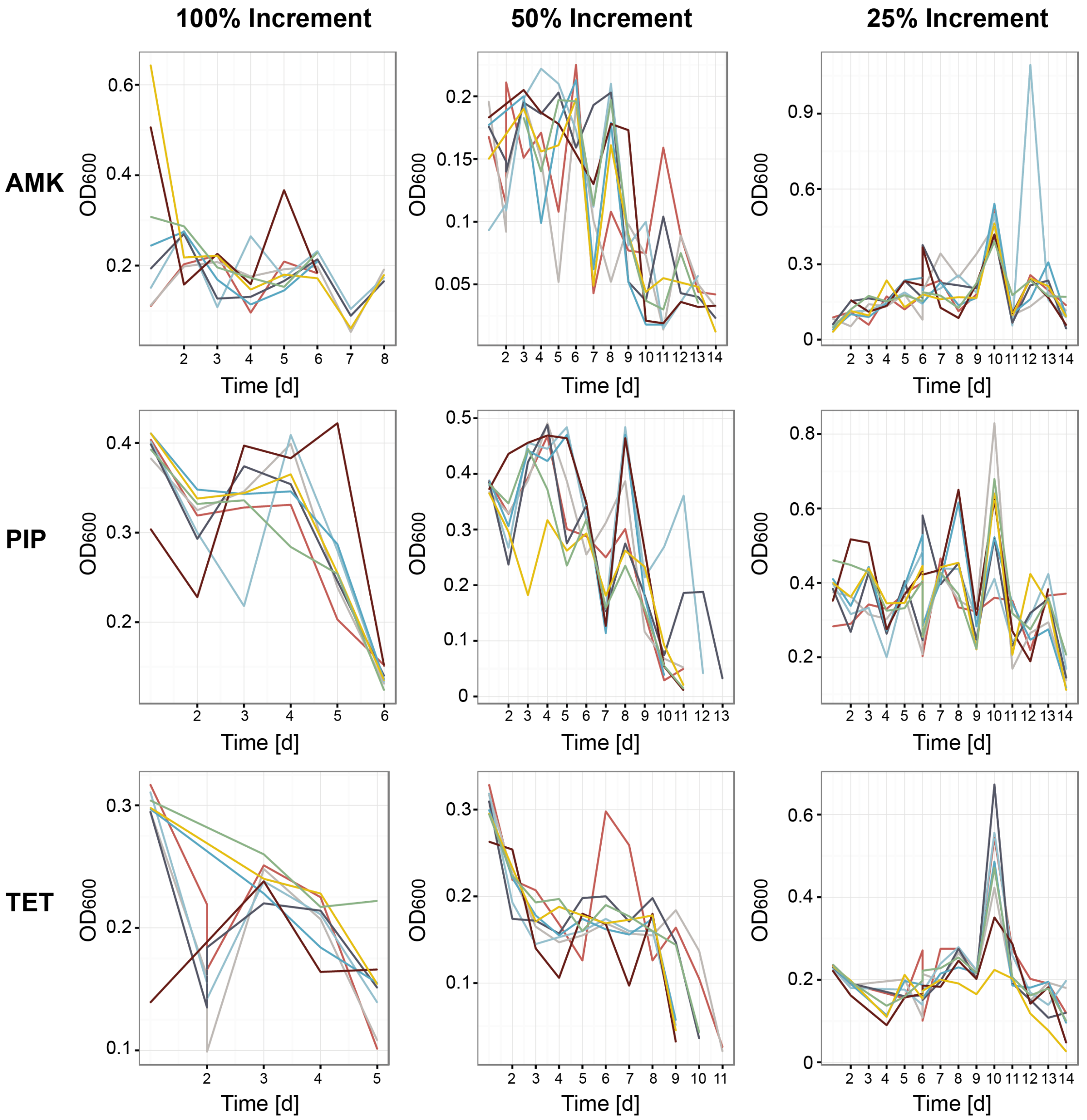

SI Figure 3: OD_{600} of increment lineages before daily transfer. The OD_{600} is displayed for all increment lineages grouped by experimental setup (25, 50 and 100 % increments) and the three drugs they have been adapted to: amikacin (AMK), piperacillin (PIP) and tetracycline (TET). In most cases the OD_{600} declined before extinction of the lineages. The different colors represent the eight different replicates.

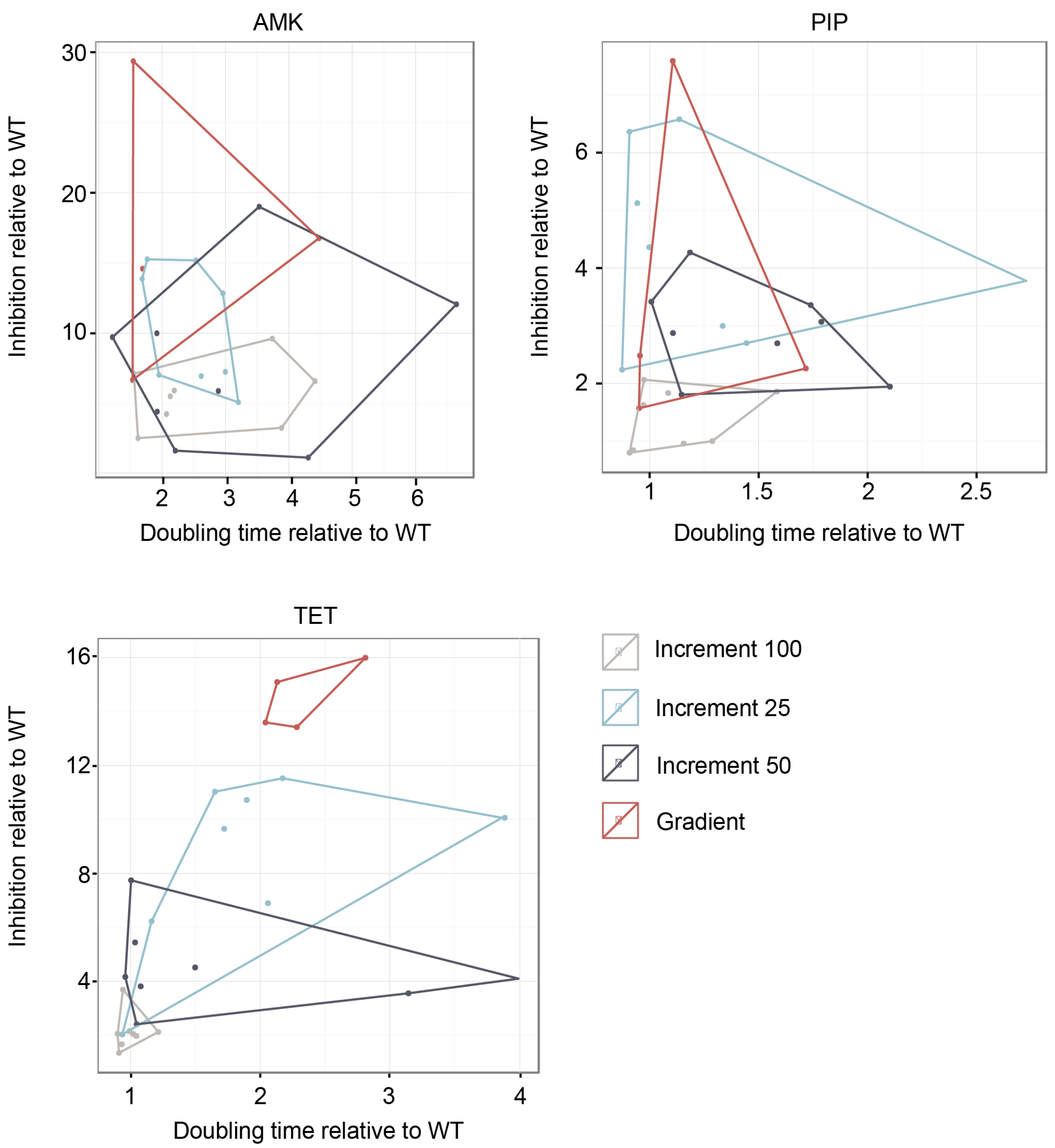

SI Figure 4: IC_{85} relative to the wild type compared to the doubling time relative to the wild type. The three plots are divided by the three drugs amikacin (AMK), piperacillin (PIP) and tetracycline (TET). The different colors represent the different experimental setups. Strains are plotted according to their relative resistance compared to the wild type (WT) and their relative doubling time. The strains marking the outer area of all strains belonging to one experimental setup are connected. No distinct correlation between high resistance with longer doubling time and low resistance with shorter doubling time can be identified.

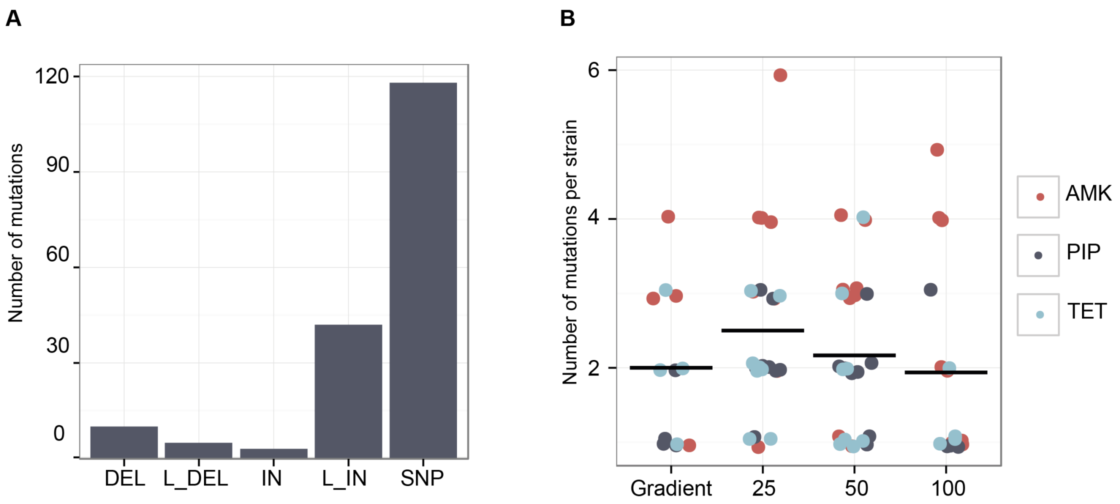

SI Figure 5: Meta-analysis of the sequencing data. (A) Number of different mutation types, discriminating between deletions (DEL), large deletions (L_DEL), insertions (IN), large insertions (L_IN) and single nucleotide polymorphisms (SNP). (B) Number of mutations per strain discriminating the lineages in color by the drugs they have been adapted to (amikacin (AMK), piperacillin (PIP) and tetracycline (TET)). According to a t.test no significant (*P* > 0.5) difference can be detected between the experiments.

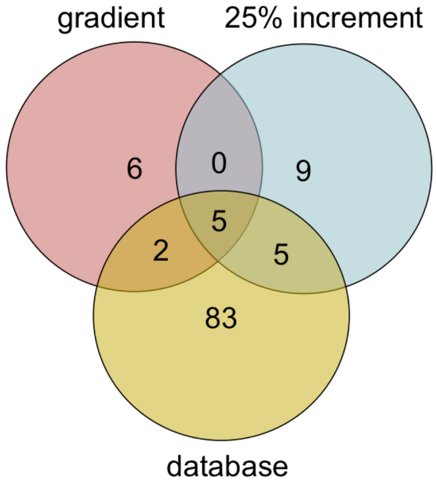

SI Figure 6: Overlap of mutated genes between the gradient evolved lineages, the clones adapted to 25 % increments and a database. In total 13 different genes have been found to be mutated in clones evolved in the gradient system and 19 different genes were mutated in lineages adapted with the 25 % increments. 90 genes were found to be mutated in 5 % of sequenced clinical *E. coli* strains. More than half of the mutations found in the gradient (7) and increment 25 % (10) adapted clones

overlap with the database and all genes (5) that were mutated in both approaches overlap with the database.

Table legends

SI Table 1: Plate design and drug concentrations of the gradient adaptive laboratory evolution experiment.

https://www.dropbox.com/s/mmacwahfojkgwzm/Gradient_concentrations.xlsx?dl=0

SI Table 2: OD600 values at each transfer of the gradient adaptive evolution experiment. This table contains all OD600 values and drug concentrations of the well that was chosen to inoculate a new gradient during the adaptive laboratory evolution experiment.

https://www.dropbox.com/s/esgdsc2mqyl54vu/OD_values_at_each_transfer.xlsx?dl=0

SI Table 3: Drug concnetrations of the increment approaches. This table gives the drug concentrations of each antibiotic for the adaptive laboratory evolution experiment for the three increment approaches.

https://www.dropbox.com/s/xgvp3rpcyxw23kp/Increment_concnetrations.xlsx?dl=0

SI Table 4: Sequencing data analysis. The table contains information about the average coverage, quality (phred score) and mapping properties of the sequencing data for every strain.

https://www.dropbox.com/s/y2ifxkq2zok81ez/Sequencing_analysis.xlsx?dl=0

SI Table 5: List of all mutations identified in the sequenced strains. Information about all mutations identified in the sequenced strains, including position of the mutation, frequency, type of mutation, annotated gene, coverage and a reference explaining the potential role in antibiotic resistance, is listed in the table.

https://www.dropbox.com/s/vxzyioycy9uq48q/Genotypic_changes.xls?dl=0
